# The complete plastid genome of *Vanda xichangensis* (Orchidaceae, Aeridinae)

**DOI:** 10.1080/23802359.2019.1688702

**Published:** 2019-11-12

**Authors:** Ding-Kun Liu, Xiong-De Tu, Sai Zhang, Ming-He Li

**Affiliations:** a Key Laboratory of National Forestry and Grassland Administration for Orchid Conservation and Utilization at College of Landscape Architecture, Fujian Agriculture and Forestry University, Fuzhou, China;; b Fujian Colleges and Universities Engineering Research Institute of Conservation and Utilization of Natural Bioresources at College of Forestry, Fujian Agriculture and Forestry University, Fuzhou, China

**Keywords:** Vandeae, Neofinetia, chloroplast genome, phylogeny

## Abstract

The complete plastid genome of *Vanda xichangensis* was determined and analyzed in this work. The plastome was 146,681 bp in length with 83,920 bp of the large single-copy (LSC) region, 11,751 bp of the small single-copy (SSC) region and 25,505 bp of the inverted repeat (IR) regions. The genome contained 120 genes, 74 protein-coding genes, 38 tRNA genes, and 8 rRNA genes. Phylogenetic analysis suggested *V. xichangensis* is sister to *V. richardsiana* plus *V. falcata*.

*Vanda* is one of the five most horticulturally important orchid genera in the world (Gardiner et al. 2013), including approximately 75 species of epiphytic or occasionally lithophytic herbs (Pridgeon et al. 2014). *Vanda xichangensis* (Z.J. Liu and S.C. Chen) L.M. Gardiner morphologically resembles *V. richardsiana*, but it can be easily distinguished by having the pale pink petals and sepals, sepals lateral pointed tip, foot curved and horizontally spreading (Liu and Chen 2004). *Vanda xichangensis*, together with the other two species, *V. falcata* and *V. richardsiana*, previously formed the genus *Neofinetia* (Gardiner 2012). Based on the nrITS, *matK*, *trnL-F*, *PsbA-trnH*, and *trnL-F*, the phylogenetic analyses showed that *Neofinetia* is sister to the remaining members of *Vanda* (Zou et al. 2016). The present study assembled the plastid genome and analysed the genome feature and phylogenetic position of *V. xichangensis*.

Fresh leaf sample of *V. xichangensis* was acquired from Xichang City (27°49′N 102°16′E), Liangshan Yi Autonomous Prefecture, Sichuan Province of China, and voucher specimen was deposited at The Orchid Conservation and Research Center of Shenzhen, Guangdong Province of China (specimen code Z.J. Liu 2747). DNA extraction from fresh leaf tissue, with 400 bp randomly interrupted for library construction. The constructed library was sequenced PE150 by Illumina Hiseq 4000 platform, approximately 20GB data generated. Illumina data were filtered by script in the cluster (default parameter: –L 5, –p 0.5, –N 0.1). Paired reads were removed when N content in sequencing reads exceeded 10% of the read base number and the low-quality (Q < =5) base number in sequencing reads exceeded 50% of the read base number (Yan et al. 2013). Complete plastid genome of *V. falcata* (KT726909) as reference, the paired-end reads were filtered with GetOrganelle pipe-line (https://github.com/Kinggerm/GetOrganelle) to get plastid-like reads, then the filtered reads were assembled by SPAdes version 3.10 (Bankevich et al. 2012), the final ‘fastg’ were filtered by the script of GetOrganelle to get pure plastid contigs, and the filtered De Brujin graphs were viewed and edited by Bandage (Wick et al. 2015). Then we can get the circle plastomes. Assembled plastid genome annotation based on comparison with *V. falcata* by GENEIOUS v11.1.5 (Biomatters Ltd., Auckland, New Zealand) (Kearse et al. 2012). The phylogeny based on the complete plastid genome shared by Aeridinae species was inferred from the ML search and ML bootstrap analysis using RAxML (Stamatakis 2014); 20 representative species of Aeridinae were aligned using MAFFT v7.307 (Katoh and Standley 2013); bootstrap probability values were calculated from 1000 replicates; *Phalaenopsis equestris* (JF719062) and *Thrixspermum japonicum* (KX871234) served as the outgroup.

The complete plastid genome sequence of *V. xichangensis* (GenBank accession MN581727) was 146,681 bp in length, with a large single-copy (LSC) region of 83,920 bp, a small single-copy (SSC) region of 11,751 bp, and a pair of inverted repeat (IR) regions of 25,505 bp. The complete genome GC content was 36.6% (LSC, 33.8%; SSC, 27.7%; IR, 43.2%) and the plastome contained 120 genes, 74 protein-coding genes, 38 tRNA genes, and 8 rRNA genes. The ML tree showed that the *V. xichangensis* is sister to *V. falcata* plus *V. richardsiana* with strong support (Figure 1).

**Figure 1.**
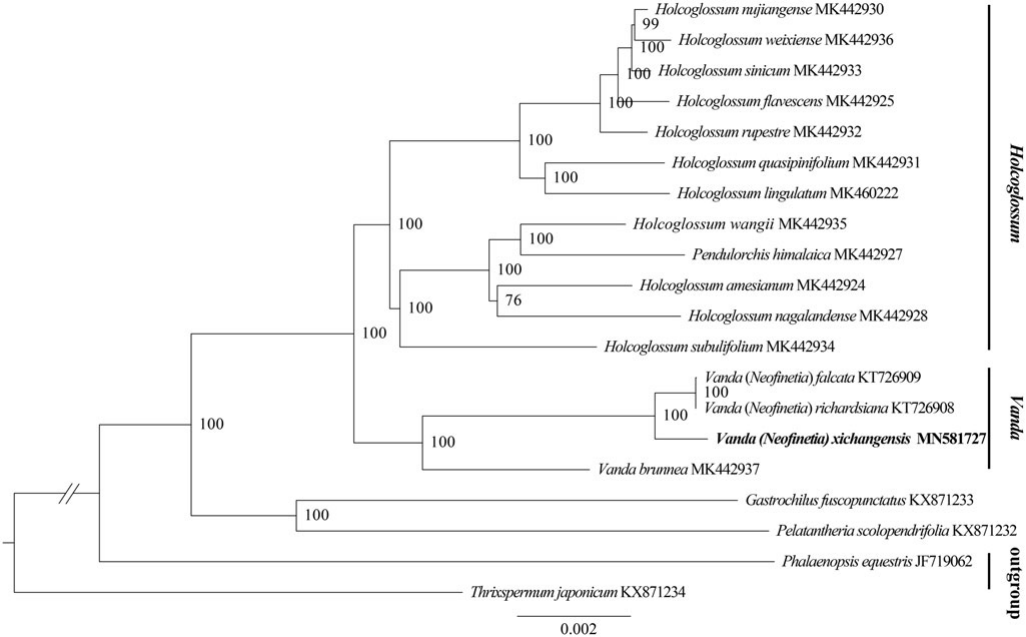
The maximum-likelihood (ML) tree based on the 20 representative plastid genomes of the subtribe Aeridinae. Numbers near the nodes mean bootstrap support value.
